# Kinetics of TH2 biomarkers in sputum of asthmatics following inhaled allergen

**DOI:** 10.3402/ecrj.v2.28319

**Published:** 2015-05-26

**Authors:** Rob G. J. A. Zuiker, Marcella K. Ruddy, Nicoletta Morelli, Robin Mogg, Veronica M. Rivas, Kristien van Dyck, Inge De Lepeleire, Michael R. L. Tanen, J. Diderik Boot, Ingrid M. C. Kamerling, Zuzana Diamant

**Affiliations:** 1 Centre for Human Drug Research, Leiden, The Netherlands; 2 EMD Serono, Rockland, MA, USA; 3 VU Medical Center, Amsterdam, The Netherlands; 4 Janssen Pharmaceutical Companies of Johnson & Johnson, Spring House, PA, USA; 5 Merck Research Laboratories, Rahway, NJ, USA; 6 Merck Research Laboratories, Brussels, Belgium; 7 HAL Allergy B.V., Leiden, The Netherlands; 8 Skane University Hospital, Department of Respiratory Medicine and Allergology, Institute for Clinical Science, Skane University, Lund, Sweden; 9 Departments of Clinical Pharmacy & Pharmacology and General Practice, University Medical Center Groningen, Groningen, The Netherlands; 10 QPS Netherlands, Groningen, The Netherlands

**Keywords:** allergen bronchial provocation test, asthma, sputum, TH2 inflammation, fluticasone

## Abstract

**Background:**

Allergen-induced late airway response offers important pharmacodynamic targets, including T helper 2 (TH2) biomarkers. However, detection of inflammatory markers has been limited in dithiothreitol-processed sputum.

**Objectives:**

To test whether allergen-induced TH2 inflammatory markers can be reproducibly quantified by sensitive detection techniques in ultracentrifuged sputum and the effect of fluticasone (FP) on these endpoints.

**Methods:**

Thirteen allergic asthmatics with dual allergen-induced airway responses, documented during a single-blind placebo run-in period, participated in a double-blind, two-period crossover study. Each period consisted of three consecutive days, separated by ≥3 weeks. Following randomization, subjects inhaled FP (500 µg bid, five doses total) or placebo. On Day 2 in each study period, allergen challenge was performed and airway response measured by forced expiratory volume in 1 sec (FEV1) until 7 h post-challenge. Sputum was induced 24 h pre-allergen and 7 and 24 h post-allergen. Sputum samples were split into two portions: TH2 biomarkers were quantified by Meso Scale multiplex platform following ultracentrifugation, and cell differentials were counted on Giemsa–May-Grünwald-stained cytospins. Allergen-induced changes in inflammatory endpoints were compared between FP and placebo using a mixed model ANCOVA.

**Results:**

Inhaled allergen induced dual airway responses in all subjects during both placebo periods with reproducible late asthmatic response (LAR) and increased sputum inflammatory biomarkers (IL-2, IL-4, IL-13, and eotaxin-1) and eosinophil counts. FP effectively blunted both the LAR and the inflammatory biomarkers.

**Conclusions:**

Combining novel, sensitive quantification methods with ultracentrifugation allows reproducible quantification of sputum biomarkers following allergen challenge, reversed by FP. This approach allows non-invasive identification of pharmacodynamic targets for anti-asthma therapies.

Inhaled allergen challenge is a highly reproducible, integral disease model enabling the investigation of several features of asthma (1). Allergen challenge can be applied to study the pathophysiology and, if complemented with (non-)invasive airway samplings, the immunobiology to allergic stimuli within the airways. In drug development, allergen challenge is an established tool predicting clinical efficacy of novel anti-allergic and anti-asthma treatments (2).

Non-invasive airway sampling by hypertonic saline-induced sputum (3) has been shown to yield reproducible increases in inflammatory cells and biomarkers following allergen-induced late asthmatic response (LAR) (4) with subsequent response to novel and existing anti-inflammatory therapies (2, 4–6). Although animal studies have provided evidence of TH2 cytokine response following allergen challenge, supported by some human studies applying bronchoscopy (7, 8), no consistent data exist on reproducible quantification of TH2 cytokines and chemokines in sputum. Accountable factors include degradation by standard sputum processing with dithiothreitol (DTT), which destroys the disulfide bounds of these inflammatory markers (9), overall low baseline concentrations, and relatively insensitive detection techniques. Some of these hurdles could be overcome by physical homogenization of sputum samples by ultracentrifugation, causing cellular disruption with subsequent release of intracellular products, in combination with sensitive detection techniques (10, 11).

Combining sputum ultracentrifugation with novel, sensitive quantification techniques using Meso Scale multi-array microplates (12) in the allergen challenge model, we aimed to study the following: 1) the feasibility of the quantification of TH2 cytokines and chemokines in sputum at 7 and 24 h post-challenge, 2) their reproducibility, and 3) their reversibility after a short course of inhaled fluticasone (FP). Furthermore, to allow comparison with other established markers of allergen-induced airway inflammation, we also measured the allergen-induced airway responses (i.e. the early asthmatic response [EAR] and LAR), exhaled nitric oxide (eNO), sputum cell differentials, and the provocative concentration of methacholine causing a fall in forced expiratory volume in 1 sec (FEV_1_) of 20% (PC_20_FEV_1_methacholine) at baseline and 24 h post-allergen, during all study periods.

## Methods

### Study population and design

**Fig. 1:**
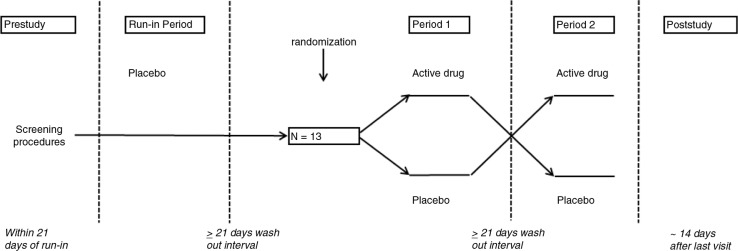
Overview of the single-blind placebo run-in period and double-blind crossover study periods 1 and 2.

**Fig. 2:**
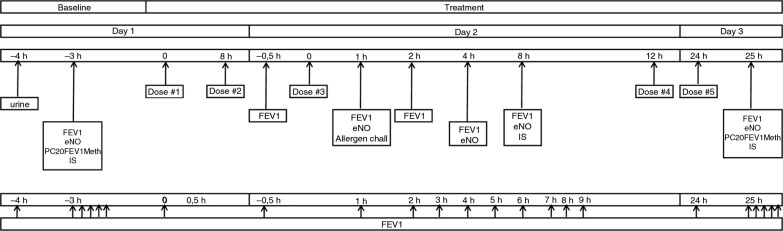
Overview of study assessments. IS, induced sputum; eNO, exhaled nitric oxide. Time zero is time of first study medication dosing. The single-blind placebo run-in screening period and the two subsequent study periods were identical.

Thirteen non-smoking subjects participated in a double-blind, two-way crossover study. Participants had clinically stable, mild to moderate allergic asthma (13), using *prn* short-acting β-2 agonists only and with dual airway responses to inhaled house dust mite (HDM), documented during the single-blind placebo run-in screening period. Each period consisted of three consecutive days, with a washout period ≥3 weeks between periods (Fig. 1). The screening, allowing to test the reproducibility of the variables, was identical to the subsequent treatment periods, during which subjects randomly received inhaled FP (metered dose inhaler [MDI], 500 µg bid, a total of five doses) or matching placebo. On Day 1, baseline measurements including eNO, spirometry followed by methacholine challenge (PC20FEV_1_methacholine), and subsequent sputum induction (3×5 min NaCl 4.5%) were performed prior to study medication. On Day 2, 1 h post-study medication, subjects underwent a titrated allergen challenge (1). The subsequent airway response was repeatedly measured by FEV_1_ up to 7 h post-allergen. eNO was measured pre-allergen and at 3 h and 7 h post-allergen, the latter measurement followed by sputum induction. At 24 h post-allergen (Day 3), test procedures were repeated as on Day 1 (Fig. 2). All test procedures were conducted according to standardized, validated methods and at the same time of the day (within 2 h) (1, 14–16).

A dual airway response to inhaled HDM extract consisted of an EAR and a LAR, defined as a fall in FEV_1_≥15% from baseline occurring between 0–3 h and 3–7 h post-allergen, respectively.

The study was approved by the Ethics Committee of Leiden University Medical Center, Leiden, The Netherlands, and all participants provided signed informed consent (EUDRACT number 2007-003671-40). All procedures were performed in accordance with the Helsinki Declaration of 1975, revised in 2008.

### Study medication and dosing rationale

FP 250 µg/puff (Allen & Hanburys, Glaxo Wellcome Ltd, Middlesex, UK) and matching placebo (Armstrong Pharmaceuticals, Inc., Canton, MA, USA, packaged at Merck Frosst, Kirkland, Canada) were supplied in identical MDIs and inhaled per single puff through an AeroChamber (Volumatic; GlaxoSmithKline, Zeist, The Netherlands). The rationale for the dose regimen was based on a previous study showing substantial reductions in allergen-induced LAR, non-specific airway hyperresponsiveness (AHR), and sputum eosinophils already following one single dose of inhaled FP 250 µg (6). Thus, to ensure optimal reversal of the allergen-induced inflammatory markers versus placebo, a total of five FP doses (500 µg per dose) were administered throughout the active treatment period.

### Allergen challenge

The allergen challenge was performed using the 2 min tidal breathing method that has been previously validated (1). The run-in period served as a dose (range)-finding procedure, whereas during the first and second study periods each subject inhaled the same two or three cumulative doses of the allergen extract that had caused a fall in FEV_1_ of at least 15% from baseline during the run-in period. Following diluent, incremental doubling concentrations (7.81–2,000 BU/mL) of HDM extract (*Dermatophagoides pteronyssinus*; SQ 503, ALK-BPT, ALK-Abelló, Almere, The Netherlands) in phosphate-buffered saline (PBS) (Invitrogen cat. no. 14040) were aerosolized by a calibrated jet-nebulizer (DeVilbiss 646, output 0.13 mL/min, Somerset, PA, USA) and inhaled at approximately 12 min intervals, until the EAR was reached (defined as a decrease in FEV_1_ of ≥15% from post-diluent baseline within 1 h post-allergen). Airway response to inhaled allergen was measured by FEV_1_ in duplicate on a calibrated spirometer (Vmax Spectra; Sensor Medics, Bilthoven, The Netherlands) according to standard procedures (17), at 10, 20, 30, 45, 60, 90, and 120 min and then hourly until 7 h after the last allergen inhalation. The highest technically valid measurement was expressed as percentage decrease from the post-diluent baseline FEV_1_ and included in the analysis.

### Methacholine challenge

The methacholine challenge was performed using standard methodologies (15). Serial doubling concentrations of methacholine bromide (MBr; Janssen Pharmaceutical, Beerse, Belgium), diluted in normal saline (NaCl 0.9%) to serial doubling dilutions of 0.15–80 µmol/mL, were aerosolized by a calibrated jet nebulizer (DeVilbiss 646) at 5-min intervals and inhaled by the subjects by tidal breathing for 2 min through the mouthpiece with the nose clipped. Airway response was measured by FEV_1_ at 30 and 90 sec (and potentially at 180 sec as well) following each concentration, and the lowest technically satisfactory FEV_1_ was incorporated into the analysis. Nebulization was continued until a ≥20% fall in FEV_1_ from the post-diluent baseline.

After both bronchoprovocation tests, subjects received salbutamol through an AeroChamber, until the FEV_1_ returned to within 10% of the baseline value.

### Exhaled nitric oxide

All eNO measurements were performed according to current guidelines (14) using a chemiluminescence analyzer (Ecomedics CLD88sp; Ecomedics, Duernten, Switzerland), which had to be replaced by a NIOX MINO^®^ (Aerocrine AB, Solna, Sweden) during the study. The NIOX MINO was used for subjects 8, 9, 10, 11, 12, and 13 during both study periods. In a previous study at our institute, both analyzers yielded similar values (18).

### Sputum induction, processing, and analysis

Sputum induction was performed as previously described (16, 19) using a DeVilbiss Ultraneb 2000 ultrasonic nebulizer (Tefa Portanje, Woerden, The Netherlands) connected to a 100-cm long plastic tube, with an internal diameter of approximately 22 mm, connected to a two-way valve (No. 2700; Hans-Rudolf, Kansas City, MO, USA) with a mouthpiece. Hypertonic saline (NaCl 4.5%) was nebulized and inhaled through the mouth, with the nose clipped, during three periods of 5 min. At approximately 7 min after each induction, spirometry was performed as a safety measure.

Collected sputum samples were divided into two portions of equal weight. The cell pellet of the first portion was processed as a full sample according to guidelines (16, 20), using 0.1% DTT (Sputolysin; Calbiochem, La Jolla, CA, USA). Cell viability and total cell count were assessed using trypan blue; sputum samples containing >80% squamous cells were excluded from analysis. Differential cell counts were performed by a qualified cytologist on May-Grünwald–Giemsa-stained, coded cytospins and expressed as a percentage of 500 nucleated, non-squamous cells.

The second sputum portion was used to quantify soluble inflammatory markers. At Merck Research Laboratories, defrosted samples were pretreated with a protease-inhibitor cocktail (50 µL per 200 mg sputum), prepared by dissolving one protease cocktail tablet (Complete Protease Inhibitor Cocktail tablets, Roche Applied Science no. 11697498001) into 50 mL of PBS. Prepared sputum samples were subsequently ultracentrifuged in an ultracentrifuge (Optima Max Ultracentrifuge, 130,000 rpm; Beckman Coulter, Inc., Fullerton, CA, USA) at 35,000 rpm (53,500×*g*) for 90 min at 4°C. Subsequently, sputum supernatant was collected and analyzed.

### Cytokine and chemokine measurements

Quantification of soluble biomarkers in sputum samples was performed using an MSD (Meso Scale Discovery, Gaithersburg, MD, USA) singleplex kit (IL-13), an MSD duplex kit (eotaxin-3 and TARC), and two MSD multiplex assays (IL-1β, IL-2, IL-4, IL-5, IL-8, IL-10, IL-12p70, IFN-γ, TNF-α, eotaxin, IP-10, MCP-1, MCP-4, and MIP-1β). All concentrations were expressed as pg/mL.

### Statistical analysis

Data from all randomized subjects were included in the analysis.

The effect of FP versus placebo on the TH2 cytokines, chemokines, and other inflammatory markers at 7 and 24 h post-allergen was assessed using a mixed-effects analysis of variance (ANOVA) model. The model included fixed factors for sequence, treatment, and period and a random effect for subjects within sequence. Between-treatment differences were estimated by the difference in least-squares means from the model with 90% confidence interval (CI; one-sided alpha=5%). Sputum cell differentials were analyzed using the actual change from baseline, whereas absolute cell counts were analyzed using the change from baseline for the square root transformed values. Geometric mean baseline sputum biomarker concentrations were calculated; half of the lower limit of quantification was used in case of negative outcomes. Changes in sputum biomarker concentrations were analyzed after log transformation and expressed as fold change from baseline.

The airway response to inhaled allergen was expressed as percentage decrease in FEV_1_ from the post-diluent baseline and plotted as time–response curves during all treatment periods. The difference in FEV_1_ during both the EAR and the LAR was analyzed using the time-weighted average of percentage change and the maximum percentage charge from baseline. Subject 1 had an initial FEV_1_ decrease of slightly under 15% at 7 h post-allergen, but met the inclusion criterion at 8 h post-allergen and was included in the study. Therefore, for this subject FEV_1_, cytokines, chemokines, and eNO were consequently measured at 8 h during all periods. FEV_1_ results at 8 h were not included in the analysis.

PC_20_FEV_1_methacholine was calculated by linear interpolation on a plot of log concentrations versus response using methacholine concentrations below and above a 20% fall in FEV_1_. The (allergen-induced and FP-reverted) changes in PC_20_FEV_1_methacholine were expressed in doubling doses. eNO was expressed as a fold change from baseline at 3, 7, and 24 h post-allergen.

Reproducibility of the allergen-induced airway responses and sputum inflammatory markers was assessed using data from the run-in and study placebo periods. The intraclass correlation coefficient (ICC) was calculated, and a two-sided paired *t*-test was performed.

### Sample size

In the absence of information about variability in TNF-α and IL-13 concentrations in sputum, eosinophil count was used as an approximate variable for sample size estimation (21). Power calculation showed that the study would have >90% power (α=0.05, one-tailed) to detect a fivefold increase from baseline at 7 h post-allergen challenge with 12 completing subjects.

## Results

### Subjects

After completion of the run-in period, 15 subjects were considered eligible. Before randomization, two subjects were withdrawn: one had a positive cotinine test, whereas the other repeatedly presented with a clinically relevant bronchoconstriction (baseline FEV_1_<70% predicted). Thus, 13 subjects were randomized and all completed the study (Table 1).

**Table 1:** Baseline characteristics of randomized subjects

Number of subjects	13
Age, years	25.9 (21–43)
Gender	4M/9F
BMI, kg/m^2^	24.4 (16.6–39.8)
FEV_1_, L	3.57 (2.92–4.50)
FEV_1_, % pred	94.0 (74.5–112.3)
PC_20_FEV_1_methacholine, µmol/mL	12.8 (0.8–81.5)
SPT HDM Wheal, mm	5.5 (2.5–10.5)
eNO, ppb	53.4 (11.2–160.8)

Numbers are expressed as mean (range). BMI, body mass index; FEV_1_, forced expiratory volume in 1 sec; SPT HDM, skin prick test for house dust mite; ppb, parts per billion.

### Safety

No serious adverse events occurred. Headache and fatigue were the most frequently reported adverse events. All events were mild in intensity and classified as unrelated to the study medication or procedures.

### Allergen-induced airway responses

**Fig. 3:**
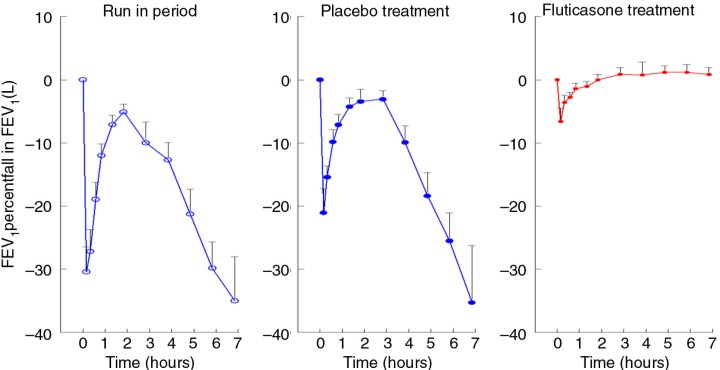
Time-response curves (mean±SEM) to inhaled allergen during run-in period, placebo treatment, and fluticasone treatment.

Inhaled HDM induced both an EAR and an LAR in all subjects during both placebo periods. Compared to placebo, FP significantly reduced the EAR and completely blunted the LAR (Fig. 3). The reproducibility of the allergen-induced LAR during both placebo periods was good, both in terms of the maximum percent fall in FEV_1_ from baseline and as time-weighted average (3–7 h post-allergen), with an ICC of 79.7 and 69%, respectively (Table 2).

**Table 2:** Analysis of the airway response to inhaled allergen

FEV_1_ endpoint	Treatment	% estimate^a^ (90% CI)	Difference in % change between fluticasone and placebo (90% CI)	One-sided *p*-value*	Reproducibility		

					Change^b^ (90% CI)	Two-sided *p*-value^c^	ICC (%)
EAR time-weighted mean	Placebo	−7.09 (−8.90, −5.28)	5.28 (3.28, 7.28)	<0.001	4.0 (0.8, 7.3)	0.020	0.0
	Fluticasone (500 mcg bid)	−1.81 (−3.62, 0.00)					
EAR maximum decrease	Placebo	−17.7 (−21.3, −14.1)	11.12 (8.26, 13.99)	<0.001	6.3 (1.0, 11.7)	0.025	21.3
	Fluticasone (500 mcg bid)	−6.56 (−10.1, −2.99)					
LAR time-weighted mean	Placebo	−13.8 (−17.0, −10.6)	15.08 (10.97, 19.18)	<0.001	2.6 (−0.8, 6.0)	0.121	69.0
	Fluticasone (500 mcg bid)	1.28 (−1.89, 4.44)					
LAR maximum decrease	Placebo	−25.9 (−31.1, −20.7)	24.00 (16.70, 31.31)	<0.001	0.0 (−4.7, 4.6)	0.982	79.7
	Fluticasone (500 mcg bid)	−1.89 (−7.06, 3.28)					
24 h	Placebo	−5.30 (−8.16, −2.44)	7.05 ( 4.45, 9.64)	<0.001	3.7 ( 0.4, 6.9)	0.030	59.4
	Fluticasone (500 mcg bid)	1.75 (−1.11, 4.61)					

a Percentage change from period baseline

b placebo period vs. (placebo) run-in period

c paired *t*-test

EAR, early asthmatic response; LAR, late asthmatic response; CI, confidence interval; ICC, intraclass correlation coefficient.

* value: fluticasone vs. placebo, one-sided alpha=5%.

### Allergen-induced non-specific AHR

**Fig. 4:**
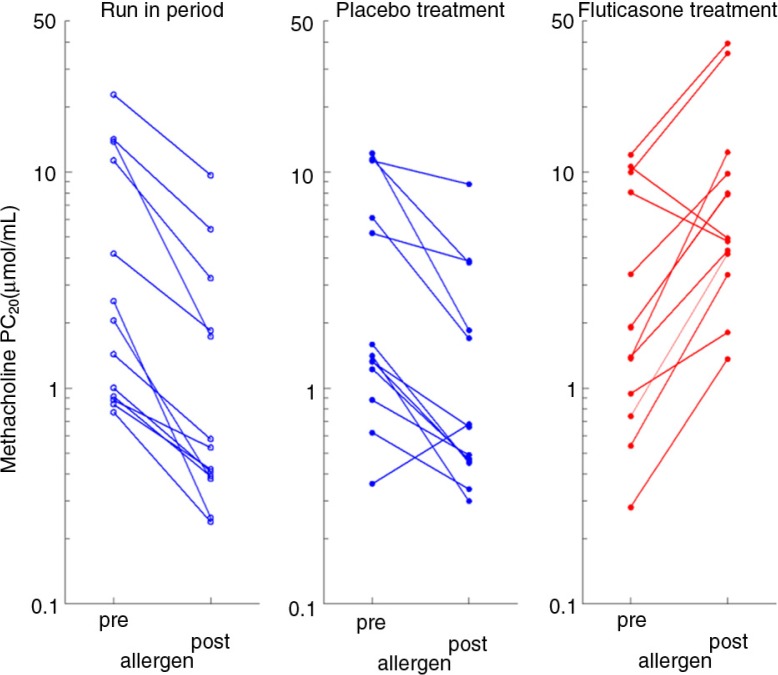
Changes in airway hyperresponsiveness 24 h pre-allergen versus 24 h post-allergen during run-in period, placebo treatment, and fluticasone treatment.

During both placebo periods, allergen challenge increased non-specific AHR, by decreasing PC_20_FEV_1_methacholine at 24 h post-allergen by, on average, 1.18 (90% CI: 1.73, 0.64) doubling doses. In contrast, FP increased 24 h post-allergen PC_20_FEV_1_methacholine by on average 1.60 doubling doses (90% CI: 1.06, 2.15), resulting in a mean difference of 2.79 doubling doses (90% CI: 2.07, 3.51; *p*<0.001) between placebo and FP (Fig. 4).

### Sputum inflammatory cells

A sputum sample was obtained from all subjects on all occasions. The average squamous cell contamination was 36% (range 2–71%). Of the 117 samples, 16 were not analyzable. Inhaled allergen significantly increased sputum eosinophils both at 7 and 24 h post-challenge during both placebo periods. This effect was significantly reduced by FP (Table 3). The reproducibility for both sputum eosinophil count (ICC 76%) and percentage (ICC 88%) was high at 7 h post-allergen, but poor (ICC 0%) at 24 h.

**Table 3:** Analysis of the eosinophils and neutrophils to inhaled allergen

Sputum differential	Time (h)	Treatment	% estimate (90% CI)^a^	% change from baseline Fluticasone vs. placebo (90% CI)	*p*-value*	Reproducibility		

						Change^b^ (90% CI)	Two-sided *p*-value^c^	ICC (%)
Eosinophils (count)	7	Placebo Fluticasone	250.8 (165.5; 336.1) −25.9 (−106; 54.5)	−277 (−394; −160)	0.002	−37.4 (−168, 93.5)	0.528	88.1
	24	Placebo Fluticasone	217.9 (142.7; 293.0) −52.9 (−128; 22.3)	−271 (−377; −165)	0.001	−118 (−244, 8.17)	0.063	0.0
Eosinophils (percent)	7	Placebo Fluticasone	11.5 (5.7; 17.4) −0.3 (−5.8; 5.2)	−11.8 (−19.9; −3.8)	0.014			
	24	Placebo Fluticasone	6.8 (1.7; 11.8) −1.1 (−6.1; 3.9)	−7.8 (−12.3, −3.3)	0.007			
Neutrophils (count)	7	Placebo Fluticasone	173.6 (62.5, 284.6) 73.0 (−31.7, 177.7)	−101 (−253, 52.0)	0.124	58.8 (−206, 324)	0.623	15.1
	24	Placebo Fluticasone	224.6 (33.1, 416.2) 35.0 (−157, 226.5)	−190 (−450, 70.6)	0.103	219 (−48.2, 487)	0.095	28.0
Neutrophils (percent)	7	Placebo Fluticasone	−5.5 (−18.8, 7.9) 0.8 (−11.9, 13.5)	6.3 (−9.3, 21.8)	0.768			
	24	Placebo Fluticasone	−5.3 (−20.9, 10.3) −9.9 (−25.5, 5.6)	−4.6 (−24.7, 15.5)	0.335			

a Sputum eosinophils and neutrophils expressed as % change from baseline at 7 and 24 h post-allergen challenge

b placebo period vs. run-in period

c paired *t*-test.

* *p*-value: fluticasone vs. placebo, one-sided alpha = 5%.

### Sputum (TH2) cytokines and chemokines

During placebo treatment, inhaled allergen increased sputum inflammatory cytokines and chemokines both at 7 and 24 h post-allergen, yielding the most robust increase at 7 h (Table 4). FP significantly blunted the allergen-induced increases in sputum concentrations of IL-5, IL-13, TARC, eotaxin-3, MCP-1, eotaxin-1, and IL-4 at 7 h post-allergen challenge and of IL-5, IL-13, eotaxin-3, IL-12p70, and MCP-1 at 24 h post-allergen challenge. None of the other sputum soluble markers were significantly affected by FP compared to placebo treatment. At 24 h post-allergen, there were no differences in any sputum inflammatory markers, with the exception of TARC between both placebo treatments.

**Table 4:** Analysis of sputum cytokines and chemokines to inhaled allergen

C	Hours post -allergen challenge	Treatment	Estimate^a^ (90% CI)	% change (fluticasone vs. placebo) (90% CI), *p*-value*	Reproducibility.		

					Change^b^ (90% CI)	Two-sided *p*-value^c^	ICC (%)
IL-5	7	Placebo Fluticasone	5.67 (3.63; 8.86) 0.99 (0.63; 1.55)	82.6 (71.4; 89.4) p<0.001	1.27 (0.53; 3.06)	0.555	48.1
	24	Placebo Fluticasone	2.57 (1.54; 4.30) 0.87 (0.52; 1.46)	66.1 (29.9; 83.6) *p*=0.011	0.69 (0.24, 2)	0.465	34.7
IL-13	7	Placebo Fluticasone	9.76 (6.25; 15.25) 1.11 (0.71; 1.73)	88.6 (81.2; 93.1) *p*<0.001	1.13 (0.63; 2.02)	0.655	73.6
	24	Placebo Fluticasone	2.11 (1.52; 2.94) 0.85 (0.61; 1.18)	59.7 (37.0; 74.2) *p*=0.002	0.66 (0.36, 1.19)	0.148	41.2
TARC	7	Placebo Fluticasone	2.13 (1.68; 2.72) 1.24 (0.97; 1.58)	41.9 (22.8; 56.3) *p*=0.003	0.9 (0.49; 1.66)	0.709	43.3
	24	Placebo Fluticasone	1.75 (1.32; 2.32) 1.37 (1.04; 1.82)	21.4 (−9.4; 43.6) *p*=0.108	0.49 (0.29, 0.82)	0.012	17.9
Eotaxin-3	7	Placebo Fluticasone	2.24 (1.71; 2.94) 1.24 (0.94; 1.64)	44.6 (18.4; 62.4) *p*=0.010	1.25 (0.55; 2.81)	0.556	0.0
	24	Placebo Fluticasone	2.56 (1.80; 3.63) 1.55 (1.09; 2.21)	39.2 (12.0; 58.0) *p*=0.018	0.73 (0.29, 1.81)	0.458	22.2
MCP-1	7	Placebo Fluticasone	1.27 (1.03; 1.56) 0.91 (0.74; 1.12)	28.2 (9.1; 43.3) *p*=0.015	1.14 (0.91; 1.44)	0.230	32.2
	24	Placebo Fluticasone	0.96 (0.81; 1.15) 0.74 (0.62; 0.89)	22.8 (4.1; 37.8) *p*=0.028	1.07 (0.82, 1.4)	0.571	8.0
Eotaxin-1	7	Placebo Fluticasone	2.01 (1.43; 2.82) 1.05 (0.75; 1.48)	47.5 (15.2; 67.6) *p*=0.018	0.68 (0.36; 1.3)	0.219	52.0
	24	Placebo Fluticasone	1.25 (0.89, 1.75) 1.26 (0.90, 1.76)	−0.7 (−61.9, 37.3) *p*=0.511	0.65 (0.26, 1.63)	0.329	0.0
IL-4	7	Placebo Fluticasone	1.52 (1.21; 1.92) 1.01 (0.80; 1.27)	34.0 (8.9; 52.2) *p*=0.021	1.02 (0.74; 1.41)	0.899	72.3
	24	Placebo Fluticasone	0.98 (0.92, 1.03) 1.02 (0.96, 1.08)	−4.7 (−13.6, 3.5) *p*=0.836	0.9 (0.72, 1.12)	0.312	0.0
MCP-4	7	Placebo Fluticasone	1.34 (0.96, 1.87) 0.91 (0.65, 1.27)	32.4 (−3.3, 55.7) *p*=0.063	1.05 (0.6; 1.85)	0.839	0.0
	24	Placebo Fluticasone	1.36 (0.98, 1.89) 1.16 (0.83, 1.61)	14.6 (−36.2, 46.5) *p*=0.277	0.87 (0.44, 1.7)	0.646	1.6
IL-12	7	Placebo Fluticasone	1.47 (0.97, 2.24) 1.05 (0.69, 1.59)	29.0 (−24.9, 59.6) *p*=0.149	1.56 (0.91; 2.68)	0.095	38.5
	24	Placebo Fluticasone	1.61 (1.07; 2.42) 0.79 (0.53; 1.19)	50.6 (12.2; 72.2) *p*=0.025	0.85 (0.46, 1.54)	0.555	26.5
IP-10	7	Placebo Fluticasone	1.11 (0.85, 1.46) 0.90 (0.69, 1.18)	19.2 (−16.6, 44.0) *p*=0.159	1.12 (0.64; 1.95)	0.671	0.0
	24	Placebo Fluticasone	1.04 (0.71, 1.53) 0.88 (0.60, 1.30)	15.4 (−42.1, 49.7) *p*=0.286	0.94 (0.52, 1.69)	0.809	0.0
MIP-1β	7	Placebo Fluticasone	1.70 (1.16, 2.48) 1.40 (0.96, 2.04)	17.8 (−40.5, 51.9) *p*=0.261	1.52 (0.66; 3.52)	0.296	14.6
	24	Placebo Fluticasone	1.70 (1.08, 2.67) 1.90 (1.21, 2.99)	−11.9 (−97.1, 36.5) *p*=0.637	0.8 (0.25, 2.55)	0.687	2.7
IL-8	7	Placebo Fluticasone	1.01 (0.80, 1.28) 0.97 (0.77, 1.23)	4.0 (−31.6, 30.0) *p*=0.409	0.93 (0.64; 1.36)	0.697	39.3
	24	Placebo Fluticasone	1.26 (0.95, 1.66) 1.15 (0.87, 1.52)	8.4 (−35.4, 38.0) *p*=0.347	0.88 (0.53, 1.46)	0.583	29.9
IL-10	7	Placebo Fluticasone	1.18 (0.94, 1.48) 1.20 (0.96, 1.50)	−1.8 (−40.0, 26.0) *p*=0.539	1.17 (0.85; 1.63)	0.300	30.5
	24	Placebo Fluticasone	1.24 (0.95, 1.61) 1.12 (0.86, 1.45)	9.7 (−17.2, 30.3) *p*=0.247	0.97 (0.61, 1.52)	0.867	11.0
IL-1β	7	Placebo Fluticasone	1.02 (0.77, 1.34) 1.12 (0.85, 1.48)	−10.6 (−60.0, 23.6) *p*=0.684	1.02 (0.64; 1.62)	0.933	42.6
	24	Placebo Fluticasone	1.17 (0.80, 1.72) 1.13 (0.77, 1.67)	3.2 (−45.1, 35.5) *p*=0.443	1.16 (0.6, 2.28)	0.628	8.9
IL-2	7	Placebo Fluticasone	0.90 (0.70, 1.16) 1.03 (0.80, 1.32)	−13.9 (−59.2, 18.5) *p*=0.752	0.81 (0.64; 1.02)	0.069	72.4
	24	Placebo Fluticasone	1.01 (0.72, 1.42) 1.21 (0.86, 1.70)	−19.6 (−79.6, 20.3) *p*=0.778	0.82 (0.42, 1.6)	0.530	13.4
IFN-γ	7	Placebo Fluticasone	1.05 (0.78, 1.43) 1.49 (1.10, 2.02)	−41.1 (−101, 1.0) *p*=0.946	1.19 (0.64; 2.23)	0.552	0.0
	24	Placebo Fluticasone	1.03 (0.77, 1.38) 1.14 (0.86, 1.53)	−10.8 (−66.7, 26.4) *p*=0.670	0.84 (0.4, 1.75)	0.611	0.0
TNF-α	7	Placebo Fluticasone	1.14 (0.88, 1.50) 1.64 (1.26, 2.15)	−43.6 (−84.1, −12.0) *p*=0.988	1.18 (0.73; 1.92)	0.460	0.0
	24	Placebo Fluticasone	1.21 (0.93, 1.56) 1.26 (0.97, 1.63)	−4.3 (−43.0, 23.9) *p*=0.593	0.86 (0.43, 1.71)	0.634	0.0

a Sputum cytokines and chemokines in fold change from baseline at 7 and 24 h post-allergen challenge after placebo and fluticasone treatment

b placebo period vs. run-in period

c paired *t*-test.

* *p*-value: fluticasone vs. placebo, one-sided alpha=5%.

At 7 h post-allergen, many soluble markers were reproducible; in particular, IL-2, IL-4, IL-13, and eotaxin-1 showed ICC values greater than 50%, with more variation between subjects than within subjects. At 24 h, none of the inflammatory markers had ICC values greater than 50%.

Cytokine baseline values on Day 1 for each treatment period are provided in Table 5.

**Table 5:** Mean* baseline values of cytokines and chemokines during placebo and fluticasone treatment

Cytokine/chemokine	IL-5	IL-13	TARC	Eotaxin-3	MCP-1	Eotaxin-1	IL-4	MCP-4	IL-12	IP-10	MIP-1β	IL-8	IL-10	IL-1β	IL-2	IFN-γ	TNF-α
Placebo Day 1 (−3 h)^a^	2.43	1.40	3.69	9.15	21.33	8.55	0.86	4.49	1.99	33.19	9.80	35.17	3.51	10.29	2.98	1.63	3.85
Fluticasone Day 1 (−3 h)^b^	2.41	1.41	3.61	8.78	22.85	9.07	0.85	5.50	2.49	39.63	8.91	32.45	3.17	8.73	2.69	1.63	3.48

a Geometric means (pg/mL)

b measured −3 h before the start of treatment on study Day 1.

### Change in eNO

Compared to baseline, eNO levels did not significantly increase at 3 and 7 h post-allergen and did not differ between placebo and FP. At 24 h post-allergen, however, a significant increase in eNO was measured (1.63-fold, 90% CI: 1.2, 2.3), which was blunted by FP (0.83-fold, 90% CI: 0.6, 1.2), resulting in a significant difference between placebo and FP of 49% (*p*=0.012, 90% CI: 19, 68).

## Discussion

In this study, we were able to reproducibly quantify several TH2 inflammatory cytokines and chemokines in sputum from allergic asthmatic subjects following inhaled allergen. The increase in these soluble sputum biomarkers was consistent with other established allergen-induced inflammatory responses and was most robust at 7 h post-allergen, coinciding with the maximal fall in FEV_1_ during the LAR. FP significantly blocked both the allergen-induced airway response and the majority of the inflammatory markers in sputum. Although other researchers previously showed a similar inflammatory response in bronchoalveolar lavage (7) and in sputum (22–24), none of them has investigated such wide range of allergen-induced TH2-cytokines and chemokines or their reversibility with corticosteroid treatment.

The use of sulfhydryl-reducing reagents, such as DTT, has complicated the detection of inflammatory cytokines and chemokines, and alternative processing techniques enabling the measurement of eotaxin, for example, have previously been published (9). In our study, sputum samples were ultracentrifuged (10), instead of being processed with DTT, to avoid potentially degrading effects on several TH2 cytokines and chemokines (9). Following this ‘boosting’ step, substantial allergen-induced increases in several cytokines and chemokines could be reproducibly quantified using sensitive detection techniques (Meso Scale multi-array microplates). However, reproducibility was lost for most soluble markers and sputum eosinophils at 24 h post-allergen.

In parallel with reproducible increases in the TH2-derived inflammatory markers, we were able to demonstrate reproducible changes in the established allergen-induced inflammatory outcome (4, 25, 26), including the late asthmatic airway response, non-specific AHR, and sputum eosinophils, underscoring the validity of our data. In agreement with previous evidence, we also found increased eNO levels at 24 h post-allergen (27), while no significant eNO increases could be observed at our cutoff point during the LAR, specifically, at 7 h post-allergen. Although previous studies showed increased eNO levels at 9 and 10 h post-allergen ((27) and (28), respectively), the present findings can be explained by the use of two different measuring devices (for logistic reasons) and the time lag required for the synthesis of inducible NO synthase, responsible for the synthesis of NO (29).

Although no direct comparison was made in the present study with soluble markers from the DTT-processed sputum portion, the current approach yielded reproducible data. In addition, the observation that FP can reverse the allergen-induced increase in these inflammatory markers in parallel with its inhibitory effects on the other inflammatory events, including the airway responses and cellular markers, suggests that this approach is sensitive enough to offer evaluation of therapeutic interventions in asthmatic subjects.

In conclusion, combining novel, sensitive quantification methods with ultracentrifugation allows reproducible quantification of sputum biomarkers following an allergen-induced LAR, which can be reversed by FP. This approach allows non-invasive identification of pharmacodynamic targets for anti-asthma therapies.
